# Poor family finances, family-based adverse childhood experiences, and depressive and behavioral symptoms in adolescence

**DOI:** 10.1007/s00127-025-02824-4

**Published:** 2025-02-07

**Authors:** Sondre Aasen Nilsen, Rebecca Lynn Radlick, Kristin Gärtner Askeland

**Affiliations:** 1 Regional Centre for Child and Youth Mental Health and Child Welfare, NORCE Norwegian Research Centre, Postbox 22 Nygårdstangen, Bergen, 5838 Norway; 2 NORCE-Norwegian Research Centre, Health & Social Sciences, Bergen, Norway

**Keywords:** Adverse childhood experiences; ACEs, Adolescence, Depressive symptoms, Behavioral problems, Family finances

## Abstract

**Purpose:**

Adverse childhood experiences (ACEs) are a possible pathway through which poor family finances influences adolescents’ behavioral and mental health problems. However, the interrelationship between family finances, ACEs, and behavioral and mental health outcomes in adolescence has received little attention. We aimed to (1) document exposure to family-based ACEs by perceived family finances (PFF), (2) examine how PFF relates to cumulative and pattern-based approaches to ACEs, and (3) assess the direct and interactive associations between PFF and ACEs in relation with behavioral and mental health problems.

**Methods:**

Data stem from the 2017–2019 nationwide Ungdata surveys of adolescents aged 13–15 (*n* = 12,560). Family-based ACEs were measured by 6 items covering parental alcohol use and intoxication, and intra-familial violence and fighting. The family-based ACEs were used both as a cumulative index score and examined through latent class analysis (LCA) to identify patterns of exposures. Cluster robust linear regression analyses were used to examine additive and interactive associations between PFF, family-based ACEs, and behavioral and depressive symptoms.

**Results:**

Poor (compared to *not poor*) PFF was significantly associated with more depressive symptoms and behavioral problems. Cumulative and pattern based approaches to family-based ACEs partially and similarly attenuated the association between PFF and mental health outcomes. Mainly, however, PFF and family-based ACEs were independently associated with mental health outcomes and did not interact.

**Conclusions:**

Poor family finances and family-based ACEs co-occur, and both have strong associations with depressive symptoms and behavioral problems in adolescence.

**Supplementary Information:**

The online version contains supplementary material available at 10.1007/s00127-025-02824-4.

## Introduction

Research has repeatedly demonstrated associations between measures of poor family finances or low socioeconomic status (SES) and emotional and behavioral mental health problems in childhood and adolescence [1, 2]. One potential pathway of influence is through the accumulation of adverse childhood experiences (ACEs) [3]. ACEs can be defined as potentially traumatic events that can negatively impact health and well-being. They include abuse (including physical-, emotional-, and sexual forms), neglect, and household or family-based challenges [see 4]. As relations and behavior within the family unit constitute an important context for youth development, the present study focuses on family-based adversities, such as parental alcohol intoxication, conflicts and violence within the family. ACEs are socially patterned, where children exposed to economic hardship or poverty are at higher risk than their more affluent peers [5–7].

The association between ACEs and mental health outcomes has been well documented [8–10]. Much of this literature has taken a cumulative approach to studying ACEs, where ACEs are added together into an index of number of exposures. A benefit of the cumulative approach is that it provides an easily quantifiable and interpretable metric of the relationship between ACEs and health outcomes. However, a drawback is that it attaches an equal weight to each ACE, meaning that no ACE is considered more or less important than others. Here, it is the number of, rather than the type and patterns of exposures, that are viewed important for child outcomes [see 11].

Latent Class Analysis (LCA) has emerged as an alternative way to model ACEs. LCA is a person-centered approach with the ability to identify unobserved latent classes based on observed data, enabling the potential to uncover distinct subgroups of individuals who share common ACE profiles [12]. In other words, LCA permits the possibility to examine whether certain patterns of exposure are more strongly related to health outcomes than others, which in turn may facilitate the examination of potential mechanisms that link ACE-exposures to child outcomes [11].

More recent studies have successfully used LCA in the context of ACEs [13]. For example, a US study of ~ 11,000 adolescents found four distinct classes (*child maltreatment*, *household dysfunction*, *community violence*, and *low adversity*), which in part, showed differential associations with anxiety, depression, and PTSD diagnosis in early adulthood [14]. This suggests that patterns of exposure matter. However, as concluded in a recent review, little is known about the predictive power of pattern-based approaches in comparison to traditional cumulative approaches [11].

Many studies include poor family finances, poverty, or other indicators of low SES as an *ACE*. However, debates have surfaced of whether this is justifiable or leads to conceptual blurring [7, 15]. According to family stress- and investment perspectives, economic hardship primarily influences children’s mental health indirectly, through the adverse effects it has on their physical and psychosocial environments [16]. Thus, if the association between social inequality and child outcomes partly operates through the differential exposure to ACEs, adding poverty or similar measures to the list of ACEs might lead to the importance of socioeconomic conditions being overlooked [15]. Moreover, it may also obscure the more complex relationships that socioeconomic conditions, ACEs, and child outcomes might have [17]. For example, ACEs could have differential impact on child outcomes depending on financial background, as children from poor families may have fewer social and personal resources to cope with such events than more affluent peers. Conversely, it is also possible that children from high SES families are more affected by such events, as it may lead to a greater disruption of their otherwise stable lives [e.g., 18].

Few studies have examined such questions. A recent longitudinal study (n = 8,572) found that the number of ACEs partly mediated the association between poverty and internalizing and externalizing symptoms in adolescence [19]. However, that study did not examine any potential interactions between poverty and ACEs on mental health outcomes. A Norwegian study (*n* = 2,043) found that the number of family stressors and negative life events partly attenuated the association between low SES and mental health problems in 11–13 year olds [20]. However, no significant interaction effects between these potential adversities and SES were found. Similarly, a study from the US (*n* = 97, ages 10–15 years), did not detect significant interaction effects between SES and ACEs on internalizing and externalizing problems when considering each ACE separately [21]. However, both of these studies relied on rather small samples of low-SES youth which might have limited their ability to detect potential interaction effects. Overall, then, the interplay between ACE exposures, socioeconomic conditions, and mental health outcomes in adolescence remain largely undecided.

## Study context and aims

Similar to other high-income countries, internalizing mental health problems (such as anxiety and depressive symptoms) have increased among Norwegian adolescents over the last decade, particularly among adolescent girls [22]. However, behavioral problems appear to be lower among Norwegian children and youth compared to many other countries [23, 24]. Although the reasons for this are not entirely clear, proposed explanations include Norway’s relatively low poverty and unemployment rates, low income inequality, and generally high educational levels [23].

We focus on three aims. First, to document the distribution of family-based ACEs across perceived family finances (PFF) in a large sample of Norwegian adolescents. Second, to assess the independent and interactive associations between PFF and family-based ACEs across two domains of mental health problems in adolescence. Finally, we aim to contribute to this research area by comparing and contrasting cumulative and pattern-based approaches to ACEs, as both have their strengths and weaknesses [11]. This study focuses on family-based ACEs - a branch within the ACE literature that centers on adverse experiences within the family unit, including family dynamics and the environment in which children are raised [25, 26].

## Methods

### Procedures

Data stem from the annual Ungdata surveys, a nationwide study of adolescents in Norway at the municipality level (for more information, see www.ungdata.no/english/). Ungdata is the most comprehensive source of information on adolescent health, lifestyle, and well-being in Norway. Ungdata was first administered in 2010 and, since 2014, has been implemented for all junior high (13–15 year olds) and high school students (16–19 year olds). Since 2010, most Norwegian municipalities have participated. Ungdata consists of a standard battery of instruments and questions that are the same each year. A voluntarily battery of additional instruments are available for order by participating municipalities. The surveys are financed by the Norwegian Directorate of Health. The Ungdata surveys are administered by Norwegian Social Research (NOVA) in co-operation with regional drug and alcohol competence centers (KoRus). Access to the Ungdata surveys can be requested by applying to the Norwegian Agency for Shared Services in Education and Research (SIKT) [27].

### Study sample and representativeness

Questions about family-based ACEs were not in the standard battery of Ungdata instruments. Thus, our sample was restricted to three Ungdata waves (2017–2019) with municipalities that had ordered these questions. In this sample, most responses were collected among students aged 13–15 years. We therefore restricted our sample to this age range, resulting in a study sample of 12,560 responses from 22 municipalities. Representativeness of this sub-sample was assessed by comparing it to the rest of the junior high school sample for the same years (*N* = 150,187). As shown in Supplementary Table 1, no notable differences between these two samples were observed in terms of age and gender distribution, perceived family finances, or depressive and behavioral problems. The distribution of individual ACEs was also similar when compared to municipalities that had included some but not all of the ACE measures. The main differences between the two samples were that the youth in the study sample had slightly more mothers and fathers with higher education, and that most responses in the included sample were collected from Eastern Norway. From 2015 to 2019, the response rate ranged from 77 to 82% among junior high school students.

### Ethical considerations

The study followed the Declaration of Helsinki guidelines. The data were provided to us in anonymized form, and all data handling and analyses have been conducted on the Secure Access to Research Data and E-infrastructure (SAFE) server provided by the University of Bergen (https://www.uib.no/en/foremployees/131011/safe). This manuscript does not contain clinical studies or patient data.

## Measures

### Age and gender

Gender was measured by adolescent self-report with response options “Boy” or “Girl.” Due to anonymity concerns in small municipalities, age was not explicitly assessed in the Ungdata surveys. In Norway, school grade placement is organized by birth cohort; therefore, we used school grade as a close proxy for age, with grades 8 through 10 (junior high school) corresponding to ages 13, 14, and 15.

### Parental education

Maternal and paternal education were assessed separately by adolescent self-report, by asking whether their mother and father had completed any higher education (at university/university college) or not, resulting in two binary items for maternal and paternal education.

### Perceived family finances (PFF)

PFF was assessed by a single item assessing whether their family had good or poor finances the last two years, with the response options: (1) *We have had good finances all the time*; (2) *We have mostly had good finances*; (3) *We have neither had good nor poor finances*; (4) *We have mostly had poor finances*; (5) *We have had poor finances all the time*. For the purpose of this study, we created a new two-level variable contrasting those with *poor perceived family finances* (collapsing response options *mostly poor* and *always poor*; coded 1), and *not poor perceived family finances* (the rest; coded 0).

### Family-based adverse childhood experiences (ACEs)

Six items were used to measure family-based ACEs: (1) Maternal alcohol use, (2) Paternal alcohol use, (3) Seen parents drunk, (4) Parents frequently quarrelling/fighting, (5) Adolescent frequently quarreling/fighting with parents, and (6) Violence from an adult in the family. All items were dichotomized to indicate the presence (coded 1) or absence (coded 0) of the given ACE, by the following rules: High maternal and paternal alcohol use was indicated if the adolescents reported that their parents drank alcohol many times a week or daily. Often seen parents drunk was indicated if the adolescents reported that they had often seen their mother or father drunk or clearly intoxicated. Parents frequently quarreling/fighting and frequently quarreling/fighting with parents was indicated if the adolescents responded *quite true* or *very true* to statements about frequent quarreling between adults in the family and with parents. Violence from an adult in the family was indicated if the adolescents reported that an adult in the family during the past 12 months had deliberately hit them one or more times. An overview of these items are shown in Supplementary Table 2.

### Depressive symptoms

Depressive symptoms were measured by Kandel and Davies’s six-item Depressive Mood Inventory [DMI; 28], derived from the Hopkins Symptom Checklist [29]. The DMI measures self-reported depressive symptoms during the preceding week. The items are rated on a four-point scale from (1) ‘Not been affected at all’ to (4) ‘Been affected a great deal’. Items are averaged to produce a mean score ranging from 1 to 4. A previous study based on data from Ungdata 2010 to 2019, found support for the measure being unidimensional and scalar measurement invariant survey years [30]. On the current study sample, ordered confirmatory factor analyses analysis indicated adequate fit of a 1-factor model based on traditional fit indices: comparative fit index [CFI] = 0.995, root mean square error of approximation [RMSEA] = 0.063 (95% CI; 0.058–0.068), and high internal consistency (omega [*ω*] = 0.89: see Supplementary Table 3 for details).

### Behavioral problems

Behavioral problems were assessed by a six-item measure of rule-breaking behaviors, measuring how often the adolescent had engaged in:1) Vandalism; 2) Illegal tagging; 3) Shoplifting; 4) Avoided paying; 5) Unmonitored nighttime wandering; and 6) Truancy, during the past 12 months. Each item was rated on a 5-point scale from “never” (coded 0) to “11 times or more” (coded 5). The items were inspired by the Bergen Anti-Social Scale, and items used in the National Longitudinal Youth Survey [31] and the Ung i Norge (Young in Norway) survey [32]. A previous study found that the scale displayed strong psychometric properties in the Ungdata surveys 2017–2019 [33]. Similar results were obtained on the current study sample following ordered confirmatory factor analyses (CFI = 0.986, RMSEA [95% CI] = 0.040 [0.035–0.045], *ω* = 0.71; Supplementary Table 3).

### Statistical analyses

This study takes two approaches to modeling ACEs. For the cumulative approach, an index score of ACEs was created by adding together the individual ACEs. The index was scored into 0, 1, 2, 3, 4 and 5 or more, representing the number of ACEs experienced by each respondent.

For the pattern-based approach, latent class analysis (LCA) was used to identify latent classes of ACE exposures by iteratively testing one through six class solutions. The LCA was performed using the poLCA R-package, which estimates classes using a combination of Expectation-Maximization (EM) and Newton-Raphson algorithms to maximize the log-likelihood function of the latent class model [34]. The optimal model was selected based on entropy (with values closer to 1 denoting better class separation), Bayesian Information Criterion (BIC), Akaike Information Criterion (AIC), adjusted BIC (aBIC), consistent AIC (cAIC), and the likelihood-ratio test. The likelihood ratio values help to evaluate model fit by indicating substantial improvements with additional classes, with diminishing improvements suggesting an optimal number of classes. We chose to also report the cAIC, as it introduces stricter penalties to improve model selection consistency in larger samples [35], which may provide additional information given the fairly large sample of the present study.

Ideally, the optimal class solution has the lowest BIC, AIC, aBIC, and cAIC values, the highest entropy values, and offers conceptual interpretability. As different fit statistics may not always converge, we decided a priori to give the most weight to BIC and likelihood-ratio values for determining the best class solution [36]. To examine the local independence assumption for the preferred model, we calculated the bivariate residuals (BVRs) for each pair of the manifest indicators, using a threshold of > 3.84 as an suggested criterion of significant model misfit [37].

To address classification uncertainty in the LCA, we generated a classification uncertainty weight for each individual based on their posterior probability of class membership, which may be written as:

$$\:{\text{W}\text{e}\text{i}\text{g}\text{h}\text{t}}_{i}=P\left({\text{C}\text{l}\text{a}\text{s}\text{s}}_{i}\right)$$

where$\:{\text{W}\text{e}\text{i}\text{g}\text{h}\text{t}}_{i}$ represents the classification uncertainty weight for individual *i*, and $\:P\left({\text{C}\text{l}\text{a}\text{s}\text{s}}_{i}\right)$ is the posterior probability that individual *i* belongs to their assigned class. These weights were applied in further analyses using the latent classes as predictors, ensuring that individuals with higher certainty in their class assignment had a proportionally greater influence on the estimated associations. This approach approximates the intent of the three-step method as described by Nylund-Gibson et al. [38].

The index score and LCA-classes were next used in a set of parallel investigations: We first assessed how the index score and LCA-classes were distributed across PFF, and how depressive symptoms and behavioral problems were distributed across the index score and latent classes of ACEs. Pairwise comparisons with a Bonferroni correction to adjust for multiple testing were used for analyses comparing mental health scores between the latent classes.

Next, PFF was used together with the index score and latent classes of ACEs (in separate analyses) as predictors of depressive symptoms and behavioral problems. Three regression models were tested for each set of predictors and outcomes: We first assessed the bivariate associations between PFF, depressive symptoms, and behavioral problems (Model 1). In Model 2, we adjusted for ACEs to assess whether the association between PFF and depressive/behavioral problems attenuated. Finally (Model 3), we examined the interaction between PFF and the index score/latent classes of ACEs, to examine whether the two approaches to ACEs were differentially associated with the two outcomes depending on PFF. All models were adjusted for gender and age due to the well-documented gender differences in mental health problems in adolescence, and as mental health problems tend to increase during adolescence.

For the cumulative approach to ACEs, linear models are presented, as preliminary analysis comparing the fit of linear versus quadratic slopes showed no clear gain in fit by including a quadratic term for the cumulative ACE variable. To account for the hierarchical structure of Ungdata (with responses nested in municipality years, nested in municipalities) survey year was added as a fixed effect in the analyses, and we present cluster robust standard errors by municipality.

All analyses were conducted using R version 4.2.2 for Mac, using the following *R*-packages; *tidyverse* (data preparations and visualizations; [39]), *poLCA* (LCA-analysis; [34]), *estimatr* (cluster robust standard errors; [40]), *ggeffects* (pairwise comparisons; [41]), and *nnet* (multinominal logistic regression; [42]). Scripts reproducing the main results are openly available on the Open Science Framework (OSF; https://osf.io/8abwd/).

## Results

Sociodemographic characteristics and family-based ACEs by perceived family finances (PFF) are shown in Table 1. Exposure to all ACEs was significantly more common among adolescents experiencing poor PFF. For example, those with poor PFF were much more likely to report often seeing parents drunk (9.5% vs. 1.2%) and to have experienced violence from an adult family member (20.3% vs. 6%).

**Table 1:** Demographic characteristics and family-based adverse childhood experiences (ACEs) by perceived financial circumstances

Characteristic	Not poor, *N* = 11,905^1^	Poor, *N* = 463^1^	*p*-value^2^
Gender			0.011
Boys	5,768 (49.8%)	199 (43.7%)	
Girls	5,819 (50.2%)	256 (56.3%)	
Age			0.024
13	3,973 (33.4%)	127 (27.4%)	
14	3,853 (32.4%)	158 (34.1%)	
15	4,079 (34.3%)	178 (38.4%)	
Maternal education			< 0.001
Higher education	9,283 (85.3%)	262 (65.5%)	
Lower education	1,595 (14.7%)	138 (34.5%)	
Paternal education			< 0.001
Higher education	8,780 (81.5%)	219 (57.0%)	
Lower education	1,998 (18.5%)	165 (43.0%)	
Frequent alcohol use mother	1,192 (10.0%)	64 (13.8%)	0.008
Frequent alcohol use father	1,907 (16.0%)	125 (27.0%)	< 0.001
Often seen parents drunk	140 (1.2%)	44 (9.5%)	< 0.001
Violence from an adult family member	716 (6.0%)	94 (20.3%)	< 0.001
Parents often fighting/quarreling	531 (4.5%)	82 (17.7%)	< 0.001
Often fighting/quarreling with parents	647 (5.4%)	81 (17.5%)	< 0.001
Cumulative ACEs			< 0.001
0	8,573 (72.0%)	216 (46.7%)	
1	1,798 (15.1%)	101 (21.8%)	
2	1,168 (9.8%)	76 (16.4%)	
3	270 (2.3%)	37 (8.0%)	
4	66 (0.6%)	20 (4.3%)	
5 or more	30 (0.3%)	13 (2.8%)	

^1^n (%)

^2^Pearson’s Chi-squared test; Fisher’s exact test; Wilcoxon rank sum test

### Latent class analysis (LCA)

Model fit statistics for the 1- through 6-class solutions from the latent class analysis (LCA) are presented in Supplementary Table 4. The 4-class solution demonstrated the best fit across the BIC, AIC, aBIC, and cAIC indices. Entropy for this model was also adequate (0.73), indicating sufficient precision in class prediction. Likelihood-ratio values showed a substantial reduction up to the 4-class model, with very small improvements beyond this point, indicating diminishing returns. Additionally, the 4-class model provided distinct, interpretable classes with acceptable class distributions, supporting its selection for further analyses. All calculated bivariate residuals (BVRs) were less than 1.22 (Supplementary Table 5), suggesting the local independence assumption held for this model.

**Fig. 1:**
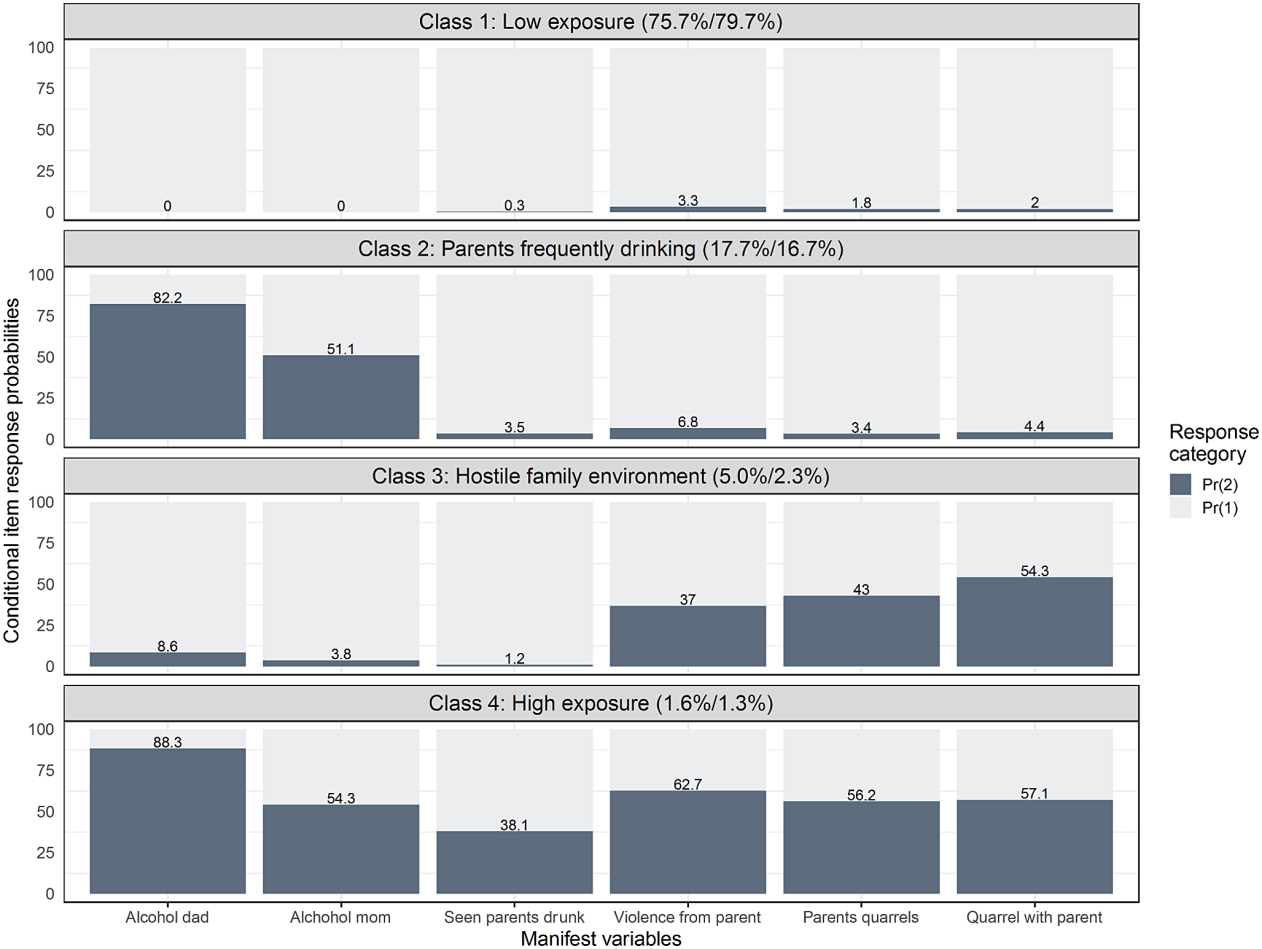
Item response probabilities across latent classes. *Note*. This figure shows the conditional item response probabilities across classes. The percentages in parenthesis behind the class-names represent the estimated population prevalence/sample prevalence

The conditional item response probabilities across the four latent classes are shown in Fig. 1. Class 1 – *Low exposure* (*n* = 10,008; 79.7% of the sample) was characterized by a low probability of all ACEs. Class 2 (*n* = 2,101; 16.7%) – *Parents frequently drinking* had high item probabilities for parents drinking many times per week (particularly the father), but low probabilities for all other ACEs, including often seeing parents drunk. Class 3 (*n* = 283; 2.3%) – *Hostile family environment* was characterized by frequent fighting/quarreling (with parents and between parents) and having experienced violence from a family member, but low parental alcohol use. Finally, Class 4 (*n* = 168; 1.3%) – *High exposure* was characterized by notable item probabilities for all ACEs.

### Distribution of adverse childhood experiences (ACEs) and mental health problems by perceived family finances (PFF)

**Fig. 2:**
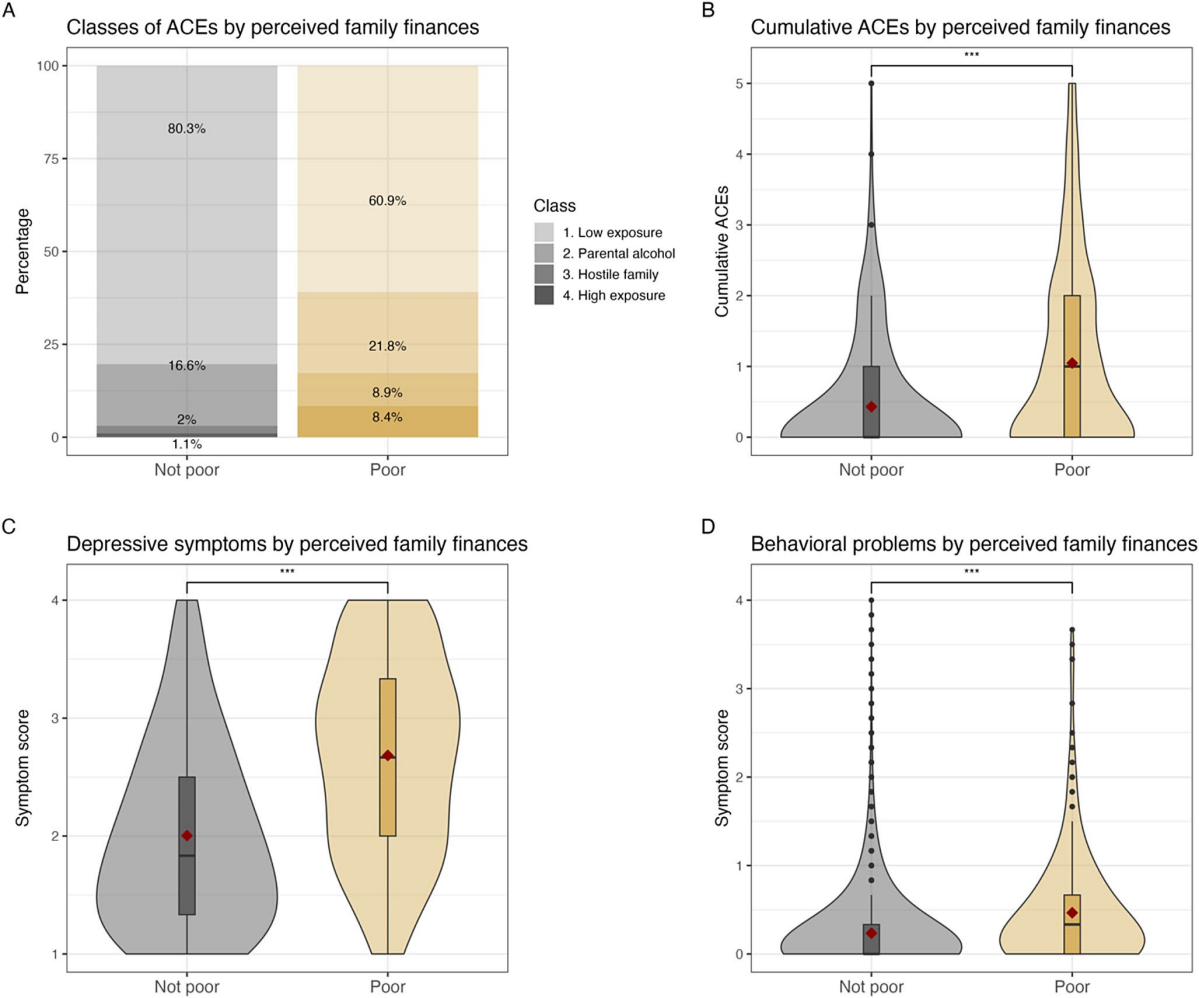
Distribution of ACEs, depressive and behavioral problems by perceived family finances. *Note*. Panel A shows a stacked bar chart of the distribution (in percentages) of latent classes of ACEs by perceived poor family finances. Panel B through Panel D shows a violin plot (density distribution) with a box plot embedded inside, of the distribution of (**B)** Cumulative ACEs by perceived family finances, (**C**) depressive symptoms by perceived family finances, and (**D**) Behavioral problems by perceived family finances. The red square denotes the mean score of each condition. *** *p* < 0.001; p-values derived from a Welch two samples t-test

The distribution of classes and cumulative ACEs by PFF are shown in Fig. 2A-B. There were more adolescents with *poor* PFF in the exposure classes (Class 2; 21.8% vs. 16.6%, Class 3; 8.9% vs. 2.0%, Class 4; 8.4% vs. 1.1%). Multinominal logistic regression analyses revealed that those with *poor* compared to *not poor* PFF had a significantly higher relative odds of being in Class 2 (OR = 1.69, 95% CI [1.32, 2.15]), Class 3 (OR = 5.77, 95% CI [3.95, 8.43]), and Class 4 (OR = 11.24, 95% CI [7.35, 17.18]), compared to being in Class 1. Likewise, those with *poor* compared to *not poor* PFF also had significantly higher number of cumulative ACEs than their peers (mean [M] = 1.05, standard deviation [SD] = 0.43, vs. M = 0.45, SD = 1.31, *p* < 0.001, p-value derived from a Welch two-sample t-test).

The distribution of depressive symptoms and behavioral problems mean scores are shown in Fig. 2C-D. Those with *poor* PFF had significantly more depressive symptoms (M = 2.68, SD = 0.83 vs. M = 2.00, SD = 0.78, Hedges’ g = 0.88, 95% CI [0.78, 0.97]) and behavioral problems (M = 0.47, SD = 0.63 vs. M = 0.24, SD = 0.41, Hedges’ g = 0.55, 95% CI [0.45, 0.64]) than peers, with medium to large standardized effect sizes.

Supplementary Fig. 1 shows the distribution of depressive symptoms and behavioral problems by ACEs. For the cumulative approach, a tendency towards a dose-response pattern was observed. For the latent classes of ACEs, there were also a general increase in symptom scores from class 1 to class 4.

### Associations between perceived family finances (PFF), adverse childhood experiences (ACEs), and mental health problems

In the regression analyses, PFF was a significant predictor of depressive symptoms and behavioral problems (see Table 2, Model 1). Youth with *poor* compared to *not poor* PFF had a higher predicted mean depression symptom score of *b* = 0.64 (95% CI; 0.56, 0.72) and behavioral problems score *b* = 0.23 (95% CI; 0.17, 0.30). In separate analyses, adjusting for the cumulative index of ACEs (Model 2a) and latent classes of ACEs (Model 2b) similarly attenuated the associations between *poor* PFF and both outcomes. However, *poor* PFF remained a significant predictor in both models. In these adjusted analyses, the cumulative index and the latent classes of ACEs were also significant predictors of depressive symptoms and behavioral problems and adding these to the models led to a notable and similar increase in the explained variance of the outcome measures. For the latent classes, all exposure classes (2–4) were associated with significantly more depressive and behavioral problems compared to class 1. Pairwise comparisons with a Bonferroni correction for multiple testing revealed significant differences between all pairs of classes for depressive symptoms. For behavioral problems, significant pairwise comparisons were detected for all pairs except between Class 3 and Class 4 (b = -0.14, 95% CI [-0.26, 0.01], *p* = 0.457; Supplementary Table 6).

**Fig. 3:**
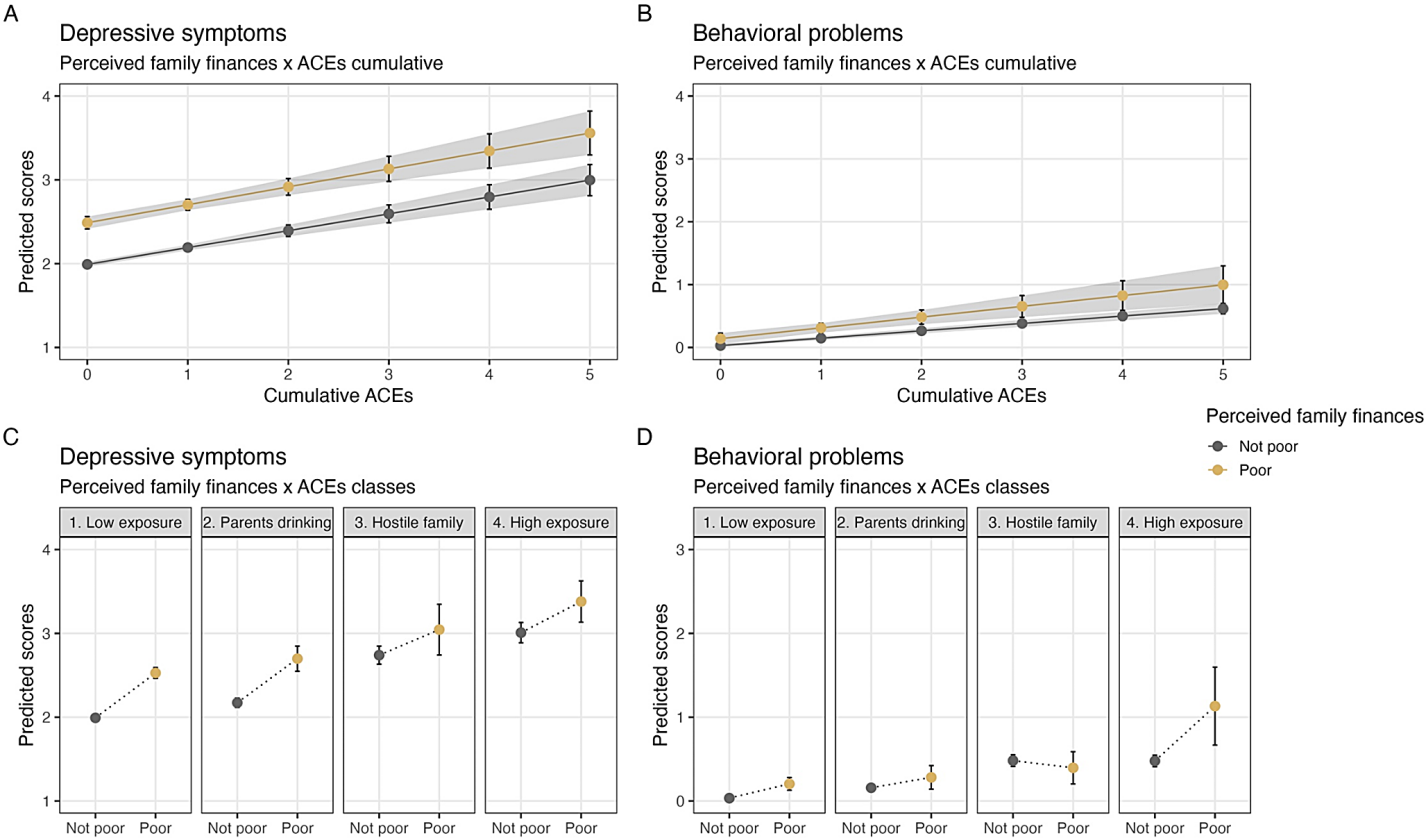
Predicted symptoms of depressive symptoms and behavioral problems by cumulative (**A**–**B**) and pattern based approaches (**C**–**D**) to family-based ACEs, by perceived family finances. *Note.* This figure shows the predicted scores of depressive symptoms and behavioral problems from the interaction models examining the interaction between perceived family finances and cumulative and pattern based approaches to family-based ACEs. Error bars represent 95% confidence intervals derived from cluster robust standard errors at the municipality level. All estimates are adjusted by age, gender, and survey year. Note that the y-axis is scaled differently across outcomes

Models 3a and 3b present the results from interaction analyses between PFF and ACEs (both cumulative and latent classes) on depressive symptoms and behavioral problems. With one exception, no significant interaction effects were detected, suggesting that the null hypothesis of no interaction effect could not be rejected. The only exception was for behavioral problems, where the association with PFF was significantly weaker among those in Class 3 vs. Class 1 (b = -0.26, 95% CI [-0.50, -0.01], *p* = 0.045). However, as shown by the wide confidence intervals, this estimate had a high degree of uncertainty (See Fig. 3).

In terms of standardized effects (here presented as predicted change in the outcome measures in standard deviation units; β), the associations between PFF, ACEs, and depressive symptoms/behavioral problems were moderate to large in size. PFF was observably more strongly associated with depressive symptoms (β = 0.81, 95% CI [0.71, 0.91]) than behavioral problems (β = 0.55, 95% CI [0.39, 0.71]). However, the associations between cumulative and latent classes of family-based ACEs were highly similar across the two outcome measures (Supplementary Tables 7–8).

**Table 2:** Associations between perceived family finances (PFF), family-based adverse childhood experiences (ACEs) and depressive symptoms and behavioral problems

	Model 1			Model 2a			Model 2b			Model 3a			Model 3b		
Depressive symptoms	b	95% CI^1^	*p*	b	95% CI^1^	*p*	b	95% CI^1^	*p*	b	95% CI^1^	*p*	b	95% CI^1^	*p*
PFF															
Not poor	Ref	Ref		Ref	Ref		Ref	Ref		Ref	Ref		Ref	Ref	
Poor	0.64	0.56, 0.72	< 0.001	0.51	0.43, 0.59	< 0.001	0.51	0.42, 0.59	< 0.001	0.50	0.42, 0.57	< 0.001	0.54	0.46, 0.61	< 0.001
ACEs cumulative				0.20	0.15, 0.25	< 0.001				0.20	0.15, 0.25	< 0.001			
ACEs classes															
Class 1 (Low)							Ref	Ref					Ref	Ref	
Class 2 (Parental alcohol)							0.18	0.11, 0.25	0.001				0.18	0.11, 0.25	0.001
Class 3 (Hostile family)							0.72	0.60, 0.83	< 0.001				0.75	0.62, 0.87	< 0.001
Class 4 (High exposure)							0.98	0.85, 1.10	< 0.001				1.00	0.87, 1.20	< 0.001
PFF x ACEs cumulative										0.01	-0.04, 0.07	0.613			
PFF x ACEs classes															
Poor * Class 2													-0.01	-0.20, 0.18	0.915
Poor * Class 3													-0.23	-0.60, 0.14	0.188
Poor * Class 4													-0.16	-0.51, 0.18	0.291
R²	0.143			0.189			0.178			0.189			0.178		
Adjusted R²	0.143			0.189			0.177			0.189			0.178		

Behavioral problems	b	95% CI^1^	*p*	b	95% CI^1^	*p*	b	95% CI^1^	*p*	b	95% CI^1^	*p*	b	95% CI^1^	*p*
PFF															
Not poor	Ref	Ref	Ref	Ref	Ref		Ref	Ref		Ref	Ref		Ref	Ref	
Poor	0.23	0.17, 0.30	< 0.001	0.16	0.09, 0.23	< 0.001	0.16	0.10, 0.23	< 0.001	0.11	0.02, 0.21	0.027	0.17	0.09, 0.25	0.001
ACEs cumulative				0.12	0.11, 0.14	< 0.001				0.12	0.10, 0.14	< 0.001			
ACEs classes															
Class 1 (Low)							Ref	Ref					Ref	Ref	
Class 2 (Parental alcohol)							0.12	0.10, 0.14	< 0.001				0.12	0.11, 0.14	< 0.001
Class 3 (Hostile family)							0.41	0.34, 0.49	< 0.001				0.45	0.37, 0.52	< 0.001
Class 4 (High exposure)							0.55	0.43, 0.67	< 0.001				0.44	0.36, 0.52	< 0.001
PFF x ACEs cumulative										0.05	-0.04, 0.15	0.214			
PFF x ACEs classes															
Poor * Class 2													-0.05	-0.21, 0.11	0.534
Poor * Class 3													-0.26	-0.50, -0.01	0.045
Poor * Class 4													0.48	-0.15, 1.10	0.110
R²	0.053			0.110			0.098			0.111			0.102		
Adjusted R²	0.053			0.109			0.098			0.110			0.101		

*Note*.^1^CI = Confidence Interval calculated using cluster-robust standard errors, clustered by municipality. Model 1: Crude association, Model 2a: Adjusted by Cumulative ACEs, Model 2b: Adjusted by latent classes of ACEs. Model 3a: Interaction model (PFF * Cumulative ACEs), Model 3b: Interaction model (PFF * ACEs classes). PFF = Perceived family finances. b = unstandardized beta coefficient. All models are adjusted for age, gender, and survey year. Models with ACE classes as predictors (2b and 3b) have been weighted by the inverse of the classification error to adjust for uncertainty in class assignment

## Discussion

We aimed to document the distribution of family-based ACEs by perceived family finances (PFF) and to assess the direct and interactive associations between poor PFF and ACEs in relation to depressive symptoms and behavioral problems in adolescence. Exposure to family-based ACEs was strongly socially patterned, with those reporting poor PFF showing higher exposure to all family-based ACEs than their peers. Family-based ACEs attenuated some of the association between poor PFF and both depressive and behavioral problems, suggesting that they could be part of the pathway through which poverty influences mental health problems. However, PFF and both cumulative and classes of ACEs were mostly independently associated with mental health problems and generally did not interact.

Adolescents with poor PFF reported more depressive symptoms and behavioral problems compared to peers from more affluent families, aligning with a large body of research [43–45]. They were also more likely to have experienced family-based ACEs, particularly violence, conflicts, and parental intoxication. This is consistent with findings from the US [5, 6] and a previous Norwegian study showing higher family stress exposures in low-SES families [20].

Taking a pattern based approach, four classes of family-based ACEs were identified: (1) *Low exposure*, (2) *Parents frequently drinking*, (3) *Hostile family environment*, and (4) *High exposure.* As research focusing on family-based ACEs is scarce and has to our knowledge not employed LCA [e.g., 26], direct comparison with other studies is complicated. However, the classes identified mimic those often reported in other studies of ACEs, in terms of identifying both low and high exposure classes [e.g., 46, 47].

Curiously, within the “Parents frequently drinking” class, high probabilities of parental drinking were accompanied by low probabilities of adolescents witnessing their parents being drunk. This may suggest a moderate yet frequent pattern of drinking, such as during meals or social gatherings, that does not reach observable levels of intoxication. Alternatively, it is possible that adolescents have a high threshold for reporting their parents as “drunk,” leading to underreporting of instances where their parents may be intoxicated.

Adolescents with poor PFF had a significantly higher odds of being in the exposure classes, and particularly in the *hostile family environment*, and the *high exposure* classes. There is a paucity of studies investigating socioeconomic inequalities in relation to patterns of ACE exposures. However, our findings are similar to those of a recent UK study, which concluded that those who had parents experiencing poverty during pregnancy were also more likely to be in the exposure classes than their peers [19].

The association between poor PFF and mental health problems partly and similarly attenuated when adjusted by cumulative ACEs and classes of ACEs. Combined, these results may suggest that family-based ACEs are one potential pathway through which poor PFF influences depressive symptoms and to a lesser extent behavioral problems. These results lend some support to the family stress model, suggesting that financial strain may influence children through parental stress, negative parenting, and interpersonal dynamics in the family [48]. In parallel, strong and direct effects of both poor PFF and ACEs remained when considered jointly. For the cumulative ACEs, each added exposure was independently associated with significantly more depressive and behavioral problems in a linear fashion, as also documented in previous research [20]. For the pattern based approach, the hostile family environment class and the high exposure class were particularly strongly and independently associated with both mental health outcomes.

We did not detect any significant interaction between PFF and the cumulative index score of ACEs on depressive symptoms and behavioral problems. For the classes of ACEs, only one interaction was statistically significant: the association between PFF and behavioral problems was weaker in Class 3 than in Class 1. On the one hand, this may suggest that experiencing hostility in the family overshadows any added effect of experiencing poor PFF on behavioral problems. However, given that this was the only significant interaction among multiple tests and had wide confidence intervals, we interpret this finding with caution. Overall, our results suggest that PFF and family-based ACEs primarily have additive associations with mental health problems in adolescence, with limited evidence of differential susceptibility based on socioeconomic standing.

Few studies have explored such interaction effects; however, our findings align with two smaller studies examining the interaction between SES and cumulative [20] and individual [21] ACEs on mental health problems in childhood and adolescence. Despite the lack of strong evidence for interaction effects, we cannot rule out their possibility, especially as our sample size may limit the precision needed to detect smaller interactions, particularly across latent classes.

The cumulative and pattern based approaches to family-based ACEs accounted for roughly equivalent amounts of variance in both outcome measures, consistent with a study on college students from the US [46]. Moreover, both approaches showed a graded association with both poor PFF and depressive and behavioral symptoms based on number of exposures. Our results thus support the notion of cumulative ACEs as predictive of mental health outcomes in adolescence. At the same time, our results align with contemporary ACE perspectives [11], that simply knowing the number of exposures offers limited guidance for appropriate interventions. That is, based on the patterns of exposure detected in this study, knowing whether the adolescent is primarily exposed to high parental alcohol use, fighting and violence in the family, or both, likely has important clinical implications for the adolescent’s and family’s needs. Assessing specific family-based ACE exposure patterns could improve the tailoring of interventions to better support these families. In this context, financial challenges in the family could also be considered, to provide more tailored support.

### Strengths and limitations

Key strengths of this study are the relatively large sample size of adolescents drawn from a nationwide study with high response rates, the use of two distinct measures of mental health problems that have been psychometrically evaluated on this specific sample, and the examination of the utility of both cumulative and pattern based approaches to ACEs, which is a stated need within this research field [11].

Several methodological limitations should be noted. First, the cross-sectional data precludes causal conclusions and inferences about the directionality of the associations studied. Although some of the included family-based ACEs, such as frequent quarrels in the family, presumably do not cause poor family finances, other ACEs, such as parental alcohol use, might. Besides the issue of directionality, the interrelationship between PFF, ACEs, and mental health problems may be more complex than we have been able to model under the conditions studied here. As implied by the causal mediation framework [49], longitudinal data could allow future studies to better parse out how PFF may impact psychological health through both direct and indirect pathways, such as for example distinguishing between mediated interaction (as when PFF both increases exposure to ACEs and modifies the effect of ACEs on mental health) from controlled direct effects (as when PFF directly impacts mental health independently of ACEs).

A second limitation was the dichotomization of PFF into “poor” versus “not-poor” groups, which loses some information about potential graded associations between PFF, ACEs, and mental health problems. However, given sample size limitations and the low prevalence of certain categories, retaining all levels would have compromised power and interpretability. Future research with larger samples may better address questions of graded associations across levels of financial difficulties.

Another limitation was the parsimonious set of family-based ACEs examined. For example, information about other potential family-based ACEs known to be associated with mental health problems in adolescence, such as parental divorce [50] parental mental and physical health problems [51, 52], and neglect [53], was not available. The inclusion of a broader set of family-based ACEs could have provided further nuances to our findings.

One more general limitation pertains to the use of self-reported data on ACEs, as there is a risk of recollection-bias. As such, the prevalence rates of each of the family-based ACEs examined in this study should be interpreted cautiously. Moreover, we cannot exclude the possibility that the degree of recollection-bias, to some extent, varies across perceived family finances. Although this may impact the precision of the estimates reported, we find it unlikely that recollection-bias acts as a sole driver of the associations reported in this study.

A final limitation pertains to the generalizability of the results, as the sample was restricted to a subset of responses from the Ungdata-surveys from municipalities that had ordered the extra battery of questions of family-based ACEs voluntarily. Although this sub-sample was quite similar to the rest of the sample in terms of sociodemographic background and rates of mental health symptoms, there may be other unmeasured selection effects. Moreover, most responses in this sub-sample were collected from Eastern Norway, meaning that the results do not necessarily generalize to the entire Norwegian population.

## Conclusions

Poor family finances and family-based ACEs go together, and both have strong associations with mental health problems in adolescence. Thus, when providing health services, care should be made to assess potential adverse family conditions experienced, particularly among adolescents from less privileged backgrounds. We further conclude that both cumulative and pattern based approaches have their merits. However, complementing cumulative measures with pattern based approaches may provide a more nuanced understanding of how ACEs co-occur and relate to mental health outcomes. Ultimately, this may aid the field as it moves towards investigating mechanisms and protective factors that may be targeted in interventions.

## Electronic supplementary material

Below is the link to the electronic supplementary material.

Supplementary Material 1

## Data Availability

Access to data supporting the results of this manuscript may be accessed by application to the Norwegian Agency for Shared Services in Education and Research (SIKT): https://sikt.no/en/tjenester/finn-data/guide-ordering-data-sikt.
