# Supplementary material for: Poor family finances, family-based adverse childhood experiences, and depressive and behavioral symptoms in adolescence
Source: Soc Psychiatry Psychiatr Epidemiol. 2025 Feb 7;60(7):1707–19. doi: 10.1007/s00127-025-02824-4 (PMC12238118; doi:10.1007/s00127-025-02824-4)
Supplement: Supplementary file 1 — Supplementary Material 1 [file 127_2025_2824_MOESM1_ESM.pdf]

Supplementary Table 1: Representativeness of the sample

	Overall, N = 162,747[1]	Excluded sample, *n* = 150,187[1]	Included sample, *n* = 12,560[1]	p-value[2]
Survey year				<0.001
2017	62,070 (38.1%)	56,249 (37.5%)	5,821 (46.3%)	
2018	39,155 (24.1%)	38,749 (25.8%)	406 (3.2%)	
2019	61,522 (37.8%)	55,189 (36.7%)	6,333 (50.4%)	
Age				<0.001
13	55,807 (34.3%)	51,613 (34.4%)	4,194 (33.4%)	
14	54,010 (33.2%)	49,935 (33.2%)	4,075 (32.4%)	
15	52,930 (32.5%)	48,639 (32.4%)	4,291 (34.2%)	
Gender				0.025
Girls	78,699 (49.6%)	72,517 (49.5%)	6,182 (50.5%)	
Geographical location				<0.001
Eastern Norway	86,538 (53.4%)	74,827 (50.0%)	11,711 (93.2%)	
Mid-Norway	12,007 (7.4%)	11,626 (7.8%)	381 (3.0%)	
Northern Norway	14,501 (8.9%)	14,439 (9.7%)	62 (0.5%)	
Southern Norway	9,101 (5.6%)	9,101 (6.1%)	0 (0.0%)	
Western Norway	40,020 (24.7%)	39,614 (26.5%)	406 (3.2%)	
Perceived family finances				0.003
Poor	6,701 (4.3%)	6,238 (4.3%)	463 (3.7%)	
Maternal education				<0.001
Lower education	27,815 (19.6%)	26,056 (20.0%)	1,759 (15.4%)	
Paternal education				<0.001
Lower education	35,135 (25.4%)	32,943 (25.9%)	2,192 (19.4%)	
Frequent alcohol use mother	3,812 (8.5%)	2,544 (7.9%)	1,268 (10.1%)	<0.001
Frequent alcohol use father	6,374 (14.6%)	4,316 (13.8%)	2,058 (16.4%)	<0.001
Violence from adult family member	7,913 (7.3%)	7,091 (7.4%)	822 (6.5%)	<0.001
Parents often fighting/quarreling	2,772 (5.4%)	2,150 (5.5%)	622 (5.0%)	0.017
Often fighting/quarreling with parents	9,653 (6.1%)	8,917 (6.1%)	736 (5.9%)	0.296
Often seen parents drunk	690 (1.6%)	505 (1.6%)	185 (1.5%)	0.387
Depressive symptoms	2.00 (0.79)	2.00 (0.79)	2.03 (0.79)	<0.001
Behavioral problems	0.25 (0.44)	0.25 (0.44)	0.24 (0.43)	0.394

[1]n (%); Mean (SD).
[2]Pearson's Chi-squared test; Wilcoxon rank sum test

Supplementary Table 2. Overview of ACE items and response options

Items	Response options
Have you ever seen your father or mother drunk or clearly intoxicated?	“No, never”; “Yes, once in a while”; “Yes, occasionally”; “Yes, often”
Has an adult in your family ever deliberately hit you?	“No, never”; “Yes, once”; “2-4 times”; “5-10 times”; “More than 10 times”
Do your parents drink alcohol? (Separate responses for mother and father)	“Yes, daily”; “Several times a week”; “Around once a week”; “Once in a while”; “No, never”
I often argue with my parents	"Not at all true"; "not very true": "Quite true"; "Very True"
The adults in my family often argue with each other	“Very true”; “Quite true”; “Not very true”; “Not true at all”

Supplementary Table 3. Model fit of the Depressive symptoms and Behavioral problems scales following ordered confirmatory factor analyses

**Model**	$\chi2$ (df)	p	CFI	RMSEA (90% CI)	TLI	SRMR
Depressive symptoms	455.573(9)	<.001	0.995	0.063 (0.058, 0.068)	0.992	0.020
Behavioral problems	185.186(9)	<.001	0.986	0.040 (0.035, 0.045)	0.977	0.038

Note. $\chi2$= chi-square goodness-of-fit based on the Satorra-Bentler correction; df = degrees of freedom; CFI; Comparative Fit Index; RMSEA = Root Mean Square Error of Approximation; CI = confidence interval. TLI = Tucker Lewis Index. SRMR= Standardized Root Mean Square Residual

Supplementary Table 4. Fit statistics from latent class analyses of two- through six-class solutions

Model	log-likelihood resid.	df	BIC	aBIC	AIC	cAIC	likelihood-ratio	Entropy
1 class	-18988.54	57	38033.70	38014.64	37989.07	38039.70	4853.03792	-
2 classes	-17104.10	50	34330.89	34289.58	34234.20	34343.89	1084.16083	0.747
3 classes	-16674.09	43	33536.95	33473.39	33388.18	33556.95	224.14743	0.786
**4 classes**	**-16588.89**	**36**	**33432.62**	**33346.82**	**33231.79**	**33459.62**	**53.75383**	**0.732**
5 classes	-16586.26	29	33493.42	33385.37	33240.52	33527.42	48.48114	0.613
6 classes	-16585.68	22	33558.32	33428.03	33253.35	33599.32	47.31631	0.401

Note. df: degrees of freedom, AIC=Akaike Information Criterion, cAIC=Consistent Akaike Criterion, BIC=Bayesian infor- mation Criterion; aBIC; adjusted Bayesian Information Criterion

Supplementary Table 5. Bivariate residuals (BVR) between pairs of ACEs indicators for the 4-class model

	Frequently drinking mother	Frequently drinking father	Violence from parent	Parents quarreling	Quarreling with parents	Seen parents drunk
Frequently drinking mother	-					
Frequently drinking father	0.003	-				
Violence from parent	0.083	0.054	-			
Parents quarreling	0.005	0.002	0.146	-		
Quarreling with parents	0.640	0.105	0.067	0.076	-	
Seen parents drunk	1.131	1.038	0.777	0.241	1.215	-

Supplementary Figure 1. Distribution of depressive symptoms and behavioral problems by cumulative (A-B) and pattern based (C-D) approaches to family-based adverse childhood conditions (ACEs).

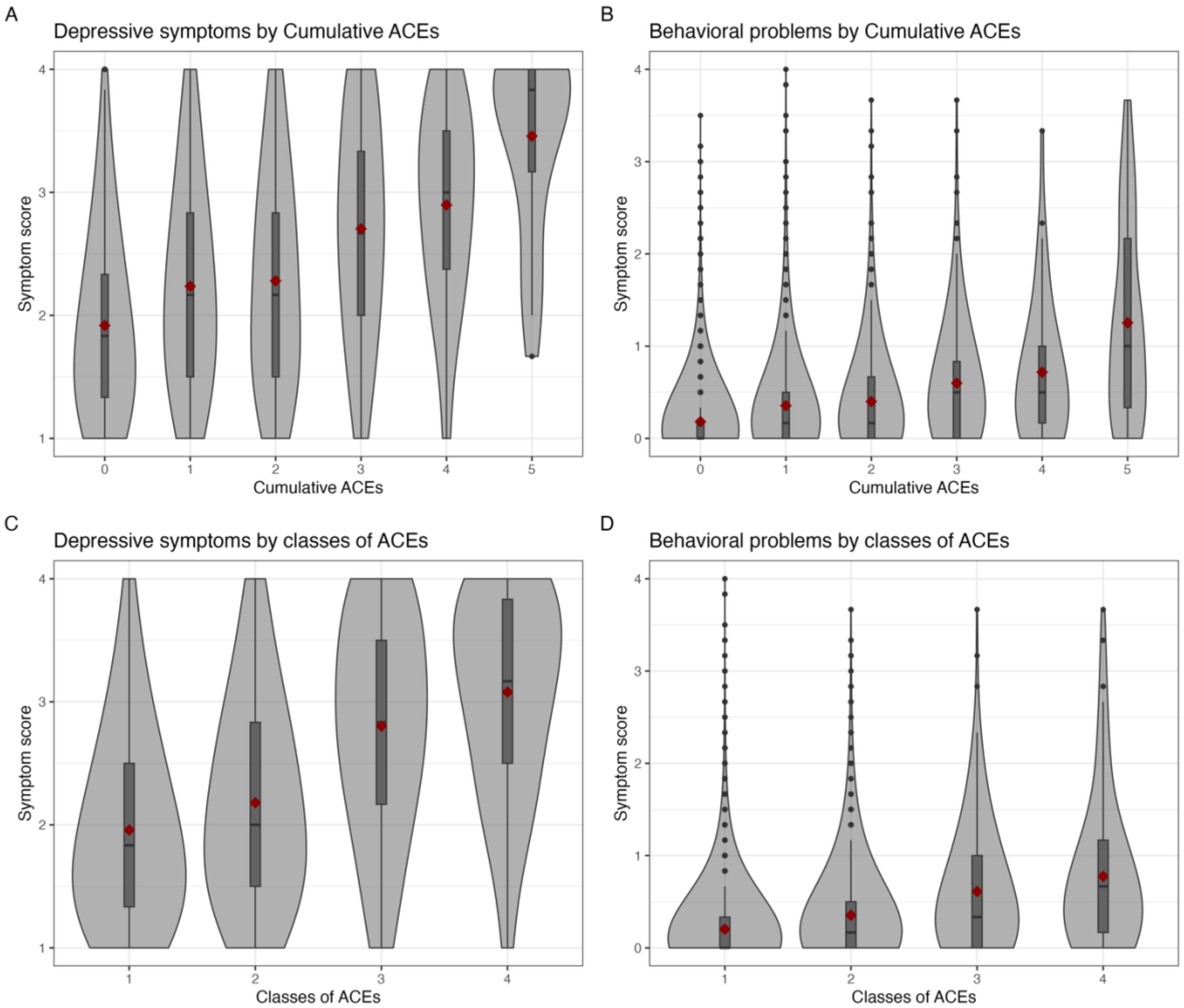

Note. This figure shows the density distribution of depressive symptoms (Panel A and C) and behavioral problems (Panel B and D) by cumulative and pattern based approaches to family-based ACEs. A boxplot is embedded within each density distribution. The red squares represent the mean scores.

Supplementary Table 6. Pairwise comparisons between classes of family-based ACEs for depressive symptoms and behavioral problems

	Depressive symptoms			Behavioral problems		
Class comparisons	b	95% CI	*p*	b	95% CI	p
1-2	-0.18	-0.24, -0.13	< .001	-0.12	-0.14, -0.10	< .001
1-3	-0.72	-0.81, -0.62	< .001	-0.41	-0.47, -0.35	< .001
1-4	-0.98	-1.09, -0.88	< .001	-0.55	-0.65, -0.45	< .001
2-3	-0.54	-0.63, -0.44	< .001	-0.29	-0.35, -0.23	< .001
2-4	-0.80	-0.92, -0.68	< .001	-0.43	-0.52, -0.34	< .001
3-4	-0.27	-0.37, -0.16	< .001	-0.14	-0.26, 0.01	0.169

Note. This table shows estimates from pairwise comparisons between all classes for depressive symptoms and behavioral problems. The pairwise comparisons are derived from Model 2b in Table 2 in the manuscript (adjusted by perceived family finances, age, gender, and survey year). 95 % CI = 95 % confidence intervals of b, derived from cluster robust (at the municipality level) standard errors. *p*-values are adjusted for multiple comparisons (separately by outcomes) using the Bonferroni correction. The models have been weighted by the inverse of the classification error to adjust of uncertainty in class assignment.

Supplementary Table 7. Standardized estimates of the associations between perceived family finances (PFF), family-based adverse childhood experiences (ACEs) and depressive symptoms

	Model 1			Model 2a			Model 2b			Model 3a			Model 3b		
Characteristic	β	95% CI[1]	*p*	β	95% CI[1]	*p*	β	95% CI[1]	*p*	β	95% CI[1]	*p*	β	95% CI[1]	p-value
Perceived family finances															
Not poor	ref.	ref.		ref.	ref.		ref.	ref.		ref.	ref.		ref.	ref.	
Poor	0.81	0.71, 0.91	<0.001	0.65	0.54, 0.75	<0.001	0.64	0.54, 0.75	<0.001	0.63	0.54, 0.73	<0.001	0.68	0.59, 0.77	<0.001
ACEs cumulative				0.26	0.19, 0.32	<0.001				0.26	0.19, 0.32	<0.001			
ACEs classes															
Class 1 (Low)							ref.	ref.					ref.	ref.	
Class 2 (Parental alcohol)							0.23	0.14, 0.32	0.001				0.23	0.14, 0.32	0.002
Class 3 (Hostile family)							0.91	0.77, 1.10	<0.001				0.95	0.79, 1.10	<0.001
Class 4 (High exposure)							1.30	1.10, 1.40	<0.001				1.30	1.10, 1.50	<0.001
PFF x ACEs cumulative										0.02	-0.06, 0.09	0.6			
PFF x ACEs classes															
Poor * Class 2													-0.01	-0.26, 0.23	>0.9
Poor * Class 3													-0.29	-0.76, 0.18	0.2
Poor * Class 4													-0.21	-0.65, 0.23	0.3
R^2	0.143			0.189			0.178			0.189			0.178		
Adjusted R^2	0.143			0.189			0.177			0.189			0.178		

Note. [1]CI = Confidence Interval calculated using cluster-robust standard errors, clustered by municipality. Model 1: Crude association, Model 2a: Adjusted by Cumulative ACEs, Model 2b: Adjusted by latent classes of ACEs. Model 3a: Interaction model (PFF * Cumulative ACEs), Model 3b: Interaction model (PFF * ACEs classes). PFF = Perceived family finances. β = semi-standardized coefficients (outcome measure has been z-transformed). All models are adjusted for age, gender, and survey year. Models using ACE classes as predictors (2b and 3b) have been weighted by the inverse of the classification error to adjust for uncertainty in class assignment.

Supplementary Table 8. Standardized estimates of the associations between perceived family poverty (PFP), family-based adverse childhood experiences (ACEs) and behavioral problems

	Model 1			Model 2a			Model 2b			Model 3a			Model 3b		
Characteristic	β	95% CI[1]	*p*	β	95% CI[1]	*p*	β	95% CI[1]	*p*	β	95% CI[1]	*p*	β	95% CI[1]	*p*
Perceived family poverty															
Not poor	ref.	ref.		ref.	ref.		ref.	ref.		ref.	ref.		ref.	ref.	
Poor	0.55	0.39, 0.71	<0.001	0.38	0.21, 0.55	<0.001	0.39	0.23, 0.55	<0.001	0.26	0.04, 0.48	0.027	0.40	0.20, 0.60	0.001
ACEs cumulative				0.29	0.25, 0.32	<0.001				0.28	0.23, 0.32	<0.001			
ACEs classes															
Class 1 (Low)							ref.	ref.					ref.	ref.	
Class 2 (Parental alcohol)							0.29	0.23, 0.34	<0.001				0.29	0.25, 0.34	<0.001
Class 3 (Hostile family)							0.97	0.79, 1.10	<0.001				1.10	0.87, 1.20	<0.001
Class 4 (High exposure)							1.30	1.00, 1.60	<0.001				1.00	0.85, 1.20	<0.001
PFF x ACEs cumulative										0.13	-0.09, 0.34	0.2			
PFF x ACEs classes															
Poor * Class 2													-0.11	-0.49, 0.27	0.500
Poor * Class 3													-0.60	-1.20, -0.02	0.045
Poor * Class 4													1.10	-0.35, 2.60	0.110
R^2	0.053			0.110			0.098			0.111			0.102		
Adjusted R^2	0.053			0.109			0.098			0.110			0.101		

Note. [1]CI = Confidence Interval calculated using cluster-robust standard errors, clustered by municipality. Model 1: Crude association, Model 2a: Adjusted by Cumulative ACEs, Model 2b: Adjusted by latent classes of ACEs. Model 3a: Interaction model (PFF * Cumulative ACEs), Model 3b: Interaction model (PFF * ACEs classes). PFF = Perceived family finances. β = semi-standardized coefficients (outcome measure has been z-transformed). All models are adjusted for age, gender, and survey year. Models using ACE classes as predictors (2b and 3b) have been weighted by the inverse of the classification error to adjust for uncertainty in class assignment.
